# Transfers to metropolitan hospitals and coronary angiography for rural Aboriginal and non‐Aboriginal patients with acute ischaemic heart disease in Western Australia

**DOI:** 10.1186/1471-2261-14-58

**Published:** 2014-05-01

**Authors:** Derrick Lopez, Judith M Katzenellenbogen, Frank M Sanfilippo, John A Woods, Michael ST Hobbs, Matthew W Knuiman, Tom G Briffa, Peter L Thompson, Sandra C Thompson

**Affiliations:** 1 Western Australian Centre for Rural Health, The University of Western Australia, Crawley, Western Australia, Australia; 2 School of Population Health, The University of Western Australia, Crawley, Western Australia, Australia; 3 School of Medicine and Pharmacology, Sir Charles Gairdner Hospital Unit, The University of Western Australia, Nedlands, Western Australia, Australia

**Keywords:** Oceanic ancestry group, Ischaemic heart disease, Myocardial infarction, Healthcare Disparities, Rural Hospitals, Health Insurance, Coronary angiography

## Abstract

**Background:**

Aboriginal people have a disproportionately higher incidence rate of ischaemic heart disease (IHD) than non-Aboriginal people. The findings on Aboriginal disparity in receiving coronary artery procedures are inconclusive. We describe the profile and transfers of IHD patients admitted to rural hospitals as emergency admissions and investigate determinants of transfers and coronary angiography.

**Methods:**

Person-linked hospital and mortality records were used to identify 28-day survivors of IHD events commencing at rural hospitals in Western Australia. Outcome measures were receipt of coronary angiography, transfer to a metropolitan hospital, and coronary angiography if transferred to a metropolitan hospital.

**Results:**

Compared to non-Aboriginal patients, Aboriginal patients with IHD were more likely to be younger, have more co-morbidities, reside remotely, but less likely to have private insurance. After adjusting for demographic characteristics, Aboriginal people with MI were less likely to be transferred to a metropolitan hospital, and if transferred were less likely to receive coronary angiography. These disparities were not significant after adjusting for comorbidities and private insurance. In the full multivariate model age, comorbidities and private insurance were adversely associated with transfer to a metropolitan hospital and coronary angiography.

**Conclusion:**

Disparity in receiving coronary angiography following emergency admission for IHD to rural hospitals is mediated through the lower likelihood of being transferred to metropolitan hospitals where this procedure is performed. The likelihood of a transfer is increased if the patient has private insurance, however, rural Aboriginal people have a lower rate of private insurance than their non-Aboriginal counterparts. Health practitioners and policy makers can continue to claim that they treat Aboriginal and non-Aboriginal people alike based upon clinical indications, as private insurance is acting as a filter to reduce rural residents accessing interventional cardiology. If health practitioners and policy makers are truly committed to reducing health disparities, they must reflect upon the broader systems in which disparity is perpetuated and work towards a systems improvement.

## Background

Cardiovascular disease is a major cause of mortality in Australia [1] and although age-standardised hospitalisation and mortality rates have fallen [2], health inequalities remain amongst Aboriginal people [2-4], rural and remote residents [2,5], and lower socio-economic status (SES) groups [2,6,7]. In particular, ischaemic heart disease (IHD) is a major contributor to the substantial life expectancy gap between Aboriginal and non-Aboriginal populations, accounting for 14% of the total gap in disease burden [8]. At all levels of remoteness, Aboriginal people have a disproportionately higher incidence rate of myocardial infarction (MI) than non-Aboriginal people [5].

The findings on Aboriginal disparity in receiving coronary artery revascularisation procedures (CARP) are inconclusive with one study showing disparity in their fully adjusted model [9], whilst another showing disparity only in the model adjusted for demographics and admission hospital [10] and yet others showing no disparity in their fully adjusted models [11,12]. However, these analyses considered metropolitan and rural patients together. In Western Australia (WA), the largest and most sparsely populated Australian state, analyses should consider rural patients separately as transfer to metropolitan cardiology centres is a pre-requisite for receiving coronary artery diagnostic and intervention procedures unavailable in rural areas. The determinants of patient transfer are complex, including clinical characteristics and non-medical factors such as age, race, bed availability, insurance status, and patient’s previous negative experiences [13-16]. Thus, it is possible that failure to transfer may be partly responsible for disparities in CARP, and may support urban health practitioners’ and policy makers’ claims of no differential in the treatment of Aboriginal patients.

Our aims were to describe the profile and metropolitan transfer of IHD patients admitted to rural hospitals as emergency admissions; and to investigate determinants (including Aboriginal status) of transfers and coronary angiography, with a specific focus on MI since it is diagnosed based on symptoms, cardiac biomarkers and ECG findings and its diagnosis is likely to be consistently coded throughout the state.

## Methods

### Study cohort

**Figure 1:**
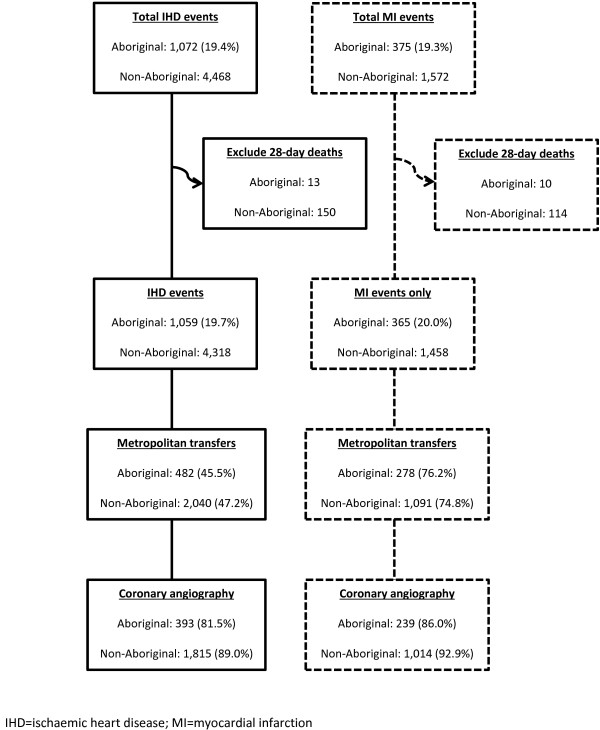
**Flow diagram of IHD and MI events from 2005-09 in rural WA by Aboriginal status.** IHD = ischaemic heart disease; MI = myocardial infarction.

We identified IHD events in rural residents aged 25–84 years who were admitted to rural hospitals in WA in 2005–09 (Figure 1). Only admissions where patients survived for more than 28 days were included to minimise likely survivor selection bias (fatal cases have less opportunity to be transferred or receive CARP). An emergency admission to a rural hospital with principal discharge diagnosis of IHD marked the starting point for each initial episode of care, defined as a series of contiguous hospital admissions, including inter-hospital transfers. An event included all admissions (booked or emergency) associated with the initial episode of care, or any additional episodes starting within a 28-day period from the initial emergency admission. The 28-day period is consistent with the International Classification of Diseases Australian Modification 10^th^ revision (ICD-10-AM) and major international studies and guidelines [17-19]. A subsequent emergency IHD admission to rural hospitals outside this event definition was considered a new event. Hence, a person could have multiple events over the study period.

### Data source

A person-linked file of all WA rural residents admitted to WA rural hospitals with principal discharge diagnosis of IHD and their subsequent hospital admissions (both rural and metropolitan) was extracted from the Hospital Morbidity Data Collection (HMDC) and Death datasets of the WA Data Linkage System [20]. At the admission date of each event, we recorded patient demographic variables, IHD category, MI type, 5-year histories of chronic pulmonary disease, diabetes, heart failure (HF) and kidney disease (ICD-10 codes defined by Quan *et al.*[21]), alcohol-related hospital admissions and smoking. Using principal diagnosis, IHD category was classified as MI (ICD-10-AM: I21), unstable angina (ICD-10-AM: I20.0) or other IHD (all other ICD-10-AM codes between I20-I25). MI type was defined as transmural (ICD-10-AM: I21.0-I21.3), subendocardial (ICD-10-AM: I21.4) or other (ICD-10-AM: I21.9). Using the Accessibility Remoteness Index of Australia Plus (ARIA+) [22], remote residence was defined as ARIA+ categories 4 (remote) and 5 (very remote) while regional included the remaining WA areas, excluding metropolitan Perth (capital of WA). Socio-Economic Indexes for Areas (SEIFA) [23] scores based on the Statistical Local Area of residence were used as a measure of SES. Private insurance status was defined as having private insurance recorded in the HMDC at any admission during the event. As Aboriginal status is under-reported in administrative health data [24,25], patients identified as Aboriginal and/or Torres Strait Islander in any admission since 1980 were classified as Aboriginal. Sensitivity analyses were performed for Aboriginal status based on identification in at least 25% of their hospital admissions or identification at the initial admission for their event.

### Study outcomes

As provision of CARP is dependent on the patient’s clinical need, suitability and consent (data not available in HMDC), coronary angiography rather than CARP was considered the main outcome, reflecting that the patient had been investigated for diagnostic and therapeutic purposes. Thus, for each event, three 28-day outcomes were identified: (i) receipt of coronary angiography (ICD-10-AM Block 668); (ii) transfer to a metropolitan hospital, defined as any admission to metropolitan hospitals during that event; and (iii) receipt of coronary angiography if transferred to a metropolitan hospital. Angiography was also assumed to have been performed if the patient had a CARP (ICD-10-AM Blocks 669–679) recorded within 28 days of the event admission date where angiography was not separately recorded in the procedure fields. For our third outcome, only rural events associated with a metropolitan transfer were included in the denominator because at the time of the study coronary angiography was performed only in seven metropolitan hospitals in WA and thus receipt of this procedure was contingent on transfer to these metropolitan hospitals. A sensitivity analysis was performed for 90-day events (deaths, transfer to a metropolitan hospital and receipt of coronary angiography within 90 days instead of 28 days) to cover unexpected delays in subsequent care.

### Statistical analyses

Analyses were performed using Stata [26]. Baseline characteristics of Aboriginal and non-Aboriginal events were summarized separately with *t*-tests and chi-squared tests used to test for significance in continuous and categorical variables respectively. To model event-based metropolitan transfers and receipt of coronary angiography, the *xtgee* command was used with a Poisson distribution for the dependent variable together with a log link function. This method accounts for repeated measures as some patients had multiple events during 2005–09. With binary outcomes, the exponentiated coefficients from Poisson regression represent risk ratios (RR) instead of incidence-rate ratios [27]. In addition to the unadjusted model that included Aboriginal status only, five models with sequential addition of variables were considered: Model 1 (age, sex); Model 2 (Model 1 + residential area, SES, IHD category/MI type); Model 3 (Model 2 + comorbidities); Model 4 (Model 3 + private insurance); and Model 5 (Model 3 with restriction to patients without private insurance).

### Ethics

Ethics approval was obtained from Human Research Ethics Committees of The University of Western Australia, WA Department of Health, WA Country Health Services and WA Aboriginal Health.

## Results

### Event characteristics

Of the 5,540 acute IHD events identified in rural WA hospitals from 2005–09 (Figure 1), 5,377 (97.1%) survived to 28 days (19.7% Aboriginal). Similar crude proportions of Aboriginal and non-Aboriginal patients with acute IHD were transferred to metropolitan hospitals, although a lower proportion of Aboriginal patients received coronary angiography if transferred (81.5% v 89.0%, p<0.001). For MI events, the proportion of Aboriginal people transferred to metropolitan hospitals was not significantly different (76.2% v 74.8%, p=0.598) although a lower proportion received coronary angiography if transferred (86.0% v 92.9%, p<0.001). Overall, similar proportions of Aboriginal and non-Aboriginal MI patients received coronary angiography (65.5% v 69.5%, p=0.134).

Compared to non-Aboriginal patients, Aboriginal patients admitted for IHD or MI events were more likely (p<0.001 mostly) to be younger, female, have more risk factors and comorbidities, and live in remote areas, but less likely to have private insurance (Table 1).

**Table 1:** Demographic and clinical profile of IHD and MI events originating from rural hospitals in WA

	**All IHD (n = 5,377 events)**			**MI only (n = 1,823 events)**		
	**Aboriginal**	**Non-Aboriginal**		**Aboriginal**	**Non-Aboriginal**	
	**n = 1,059 events (794 patients)**	**n = 4,318 events (3,587 patients)**	**p value**	**n = 365 events (342 patients)**	**n = 1,458 events (1,386 patients)**	**p value**
Age groups			<0.001			<0.001
25-34 years	53 (5.0)	31 (0.7)		25 (6.8)	10 (0.7)	
35-44 years	222 (21.0)	312 (7.2)		83 (22.7)	111 (7.6)	
45-54 years	353 (33.3)	760 (17.6)		116 (31.8)	295 (20.2)	
55-64 years	247 (23.3)	1,073 (24.8)		87 (23.8)	359 (24.6)	
65-74 years	117 (11.0)	1,051 (24.3)		36 (9.9)	333 (22.8)	
75-84 years	67 (6.3)	1,091 (25.3)		18 (4.9)	350 (24.0)	
Sex: female (%)	465 (43.9)	1,419 (32.9)	<0.001	120 (34.2)	368 (25.2)	0.001
Residential area (%)			<0.001			<0.001
Regional	424 (40.0)	3,431 (79.5)		125 (34.2)	1,128 (77.4)	
Remote	635 (60.0)	887 (20.5)		240 (65.8)	330 (22.6)	
SES (%)			<0.001			<0.001
1^st^ quartile^(a)^	576 (54.4)	1,218 (28.2)		209 (57.3)	401 (27.5)	
2^nd^ quartile	209 (19.7)	1,205 (27.9)		63 (17.3)	425 (29.1)	
3^rd^ quartile	166 (15.7)	1,388 (32.1)		50 (13.7)	465 (31.9)	
4^th^ quartile^(b)^	108 (10.2)	507 (11.7)		43 (11.8)	167 (11.5)	
Risk factors (%)						
Alcohol	311 (29.4)	234 (5.4)	<0.001	96 (26.3)	59 (4.0)	<0.001
Smoking	756 (71.4)	2,388 (55.3)	<0.001	263 (72.1)	779.(53.4)	<0.001
Comorbidities (%)						
Chronic pulmonary disease	204 (19.3)	483 (11.2)	<0.001	56 (15.3)	128 (8.8)	<0.001
Diabetes	608 (57.4)	1,113 (25.8)	<0.001	205 (56.2)	340 (23.3)	<0.001
HF	238 (22.5)	666 (15.4)	<0.001	79 (21.6)	230 (15.8)	0.008
Kidney disease	175 (16.5)	316 (7.3)	<0.001	67 (18.4)	110 (7.5)	<0.001
First ever IHD/MI event in 2005–09 (%)	591 (55.8)	3,035 (70.3)	<0.001	263 (72.1)	1,241 (85.1)	<0.001
Private insurance (%)	52 (4.9)	1,181 (27.4)	<0.001	21 (5.8)	470 (32.2)	<0.001
IHD category (%)			0.497			
MI	365 (33.8)	1,458 (33.8)				
Unstable angina	340 (32.1)	1,468 (34.0)				
Other IHD	354 (33.4)	1,392 (32.2)				
MI type (%)^(c)^						
Transmural				167 (45.8)	641 (44.0)	0.078
Subendocardial				144 (39.5)	527 (36.1)	
Other				54 (14.8)	290 (19.9)	

Statistical significance determined using t-test and chi-squared test for continuous and categorical variables respectively.

HF = heart failure; IHD = ischaemic heart disease; MI = myocardial infarction; SES = socio-economic status.

^(a)^most disadvantaged; ^(b)^least disadvantaged; ^(c)^MI type was based on ICD-10-AM coding terminology.

### Aboriginal disparity in receipt of coronary angiography

Aboriginal people with IHD were less likely to receive coronary angiography after adjusting for demographic factors, IHD category and comorbidities (Model 3: RR_IHD_ 0.87, 95% CI 0.80-0.95) but there was no significant disparity after adjusting for private insurance (Model 4) or restricting to patients without private insurance (Model 5) (Table 2). Aboriginal people with MI were also less likely to receive coronary angiography after adjusting for demographic factors and MI type (Model 2: RR_MI_ 0.81, 95% CI 0.75-0.89) but this disparity was insignificant after adjusting for comorbidities (Model 3) and private insurance (Model 4).

**Table 2:** Ratio of Aboriginal to non-Aboriginal risks of coronary angiography, transfer, and coronary angiography if transferred

	**IHD**		**MI**	
**Aboriginal status = yes**	**RR (95% CI)**	**p value**	**RR (95% CI)**	**p value**
**(i) Receipt of coronary angiography**				
Unadjusted	0.88 (0.80-0.97)	0.010	0.94 (0.87-1.02)	0.162
Model 1	0.77 (0.69-0.85)	<0.001	0.79 (0.73-0.86)	<0.001
Model 2	0.76 (0.70-0.83)	<0.001	0.81 (0.75-0.89)	<0.001
Model 3	0.87 (0.80-0.95)	0.002	0.93 (0.86-1.01)	0.096
Model 4	0.95 (0.88-1.04)	0.283	0.96 (0.88-1.05)	0.366
Model 5	0.93 (0.84-1.03)	0.166	0.97 (0.87-1.07)	0.510
**(ii) Transfer to metropolitan hospital**				
Unadjusted	0.96 (0.89-1.04)	0.366	1.02 (0.95-1.09)	0.597
Model 1	0.86 (0.79-0.94)	0.001	0.88 (0.83-0.94)	<0.001
Model 2	0.84 (0.78-0.91)	<0.001	0.89 (0.83-0.95)	0.001
Model 3	0.91 (0.84-0.98)	0.012	0.96 (0.90-1.03)	0.256
Model 4	0.99 (0.92-1.07)	0.758	0.99 (0.93-1.06)	0.804
Model 5	0.97 (0.89-1.06)	0.543	1.00 (0.92-1.08)	0.939
**(iii) Receipt of coronary angiography if transferred to metropolitan hospital**				
Unadjusted	0.92 (0.87-0.96)	<0.001	0.92 (0.88-0.97)	0.003
Model 1	0.88 (0.84-0.93)	<0.001	0.89 (0.84-0.95)	<0.001
Model 2	0.89 (0.85-0.94)	<0.001	0.91 (0.86-0.97)	0.003
Model 3	0.97 (0.92-1.02)	0.188	0.97 (0.92-1.03)	0.304
Model 4	0.98 (0.93-1.03)	0.346	0.97 (0.92-1.03)	0.382
Model 5	0.97 (0.91-1.03)	0.300	0.98 (0.91-1.05)	0.546

Model 1: age group and sex.

Model 2: model 1 + residential area, SES, IHD category (for IHD) or MI type (for MI).

Model 3: model 2 + 5-year histories of chronic pulmonary disease, diabetes, HF, kidney disease.

Model 4: model 3 + private insurance status.

Model 5: model 3 with restriction to patients without private insurance.

RR = risk ratio with reference group being non-Aboriginal patients; 95% CI = 95% confidence interval; HF = heart failure; IHD = ischaemic heart disease; MI = myocardial infarction; SES = socio-economic status.

### Aboriginal disparity in transfer to metropolitan hospital

Aboriginal people with IHD were less likely to be transferred to metropolitan hospitals after adjusting for demographic factors, IHD category and comorbidities (Model 3: RR_IHD_ 0.91, 95% CI 0.84-0.98) although this disparity was not significant after adjusting for private insurance (Model 4) or restricting to patients without private insurance (Model 5) (Table 2). Aboriginal people with MI were also less likely to be transferred to metropolitan hospitals after adjusting for demographic factors and MI type (Model 2: RR_MI_ 0.89, 95% CI 0.83-0.95); again there was no significant disparity after adjusting for comorbidities and accounting for private insurance.

### Aboriginal disparity in receipt of coronary angiography if transferred to metropolitan hospital

After adjusting for demographic factors and IHD category/MI type, Aboriginal people with IHD or MI were less likely to receive coronary angiography when transferred to a metropolitan hospital (Model 2: RR_IHD_ 0.89, 95% CI 0.85-0.94; RR_MI_ 0.91, 95% CI 0.86-0.97). Again, this disparity was not significant after adjusting for comorbidities or for private insurance (Table 2).

### Individual characteristics associated with outcomes

In the full multivariate model (presented for MI events only), factors adversely associated with receiving coronary angiography and transfer to metropolitan hospital included older age groups, chronic pulmonary disease, HF and private insurance (Table 3). However, once transferred to a metropolitan hospital, only the oldest age group (75–84 years) and those with HF or kidney disease were less likely to receive coronary angiography. A similar multivariate model but restricted to patients without private insurance resulted in similar RR (Additional file 1).

**Table 3:** Full model RR in MI patients for coronary angiography, transfer, and coronary angiography if transferred

	**Coronary angiography**		**Transfer to metropolitan hospital**		**Coronary angiography if transferred to metropolitan hospital**	
	**RR (95% CI)**	**p value**	**RR (95% CI)**	**p value**	**RR (95% CI)**	**p value**
Aboriginal status						
Non-Aboriginal	1.00		1.00		1.00	
Aboriginal	0.96 (0.88-1.05)	0.366	0.99 (0.93-1.06)	0.804	0.97 (0.92-1.03)	0.382
Age groups						
25-34 years	1.11 (0.93-1.31)	0.244	1.04 (0.91-1.20)	0.559	1.07 (0.99-1.17)	0.107
35-44 years	1.04 (0.97-1.12)	0.298	1.05 (0.98-1.12)	0.141	1.00 (0.95-1.05)	0.900
45-54 years	1.00		1.00		1.00	
55-64 years	0.98 (0.92-1.05)	0.541	1.00 (0.95-1.05)	0.959	0.98 (0.94-1.02)	0.331
65-74 years	0.86 (0.79-0.94)	0.001	0.87 (0.80-0.93)	<0.001	1.00 (0.95-1.04)	0.870
75-84 years	0.52 (0.45-0.60)	<0.001	0.59 (0.52-0.66)	<0.001	0.88 (0.82-0.96)	0.002
Sex						
Male	1.00		1.00		1.00	
Female	0.98 (0.91-1.06)	0.648	0.97 (0.91-1.03)	0.290	1.01 (0.97-1.05)	0.786
Residential area						
Regional	1.00		1.00		1.00	
Remote	1.03 (0.96-1.09)	0.488	1.06 (1.00-1.12)	0.035	0.97 (0.93-1.01)	0.147
SES quartiles						
1^st^ quartile^(a)^	1.00		1.00		1.00	
2^nd^ quartile	1.06 (0.99-1.15)	0.115	1.05 (0.98-1.12)	0.153	1.01 (0.97-1.05)	0.691
3^rd^ quartile	1.08 (1.00-1.17)	0.049	1.05 (0.99-1.13)	0.126	1.02 (0.98-1.07)	0.313
4^th^ quartile^(b)^	1.08 (0.98-1.18)	0.107	1.11 (1.04-1.19)	0.003	0.97 (0.91-1.03)	0.278
MI type^(c)^						
Transmural	1.00		1.00		1.00	
Subendocardial/other	0.96 (0.91-1.01)	0.131	0.96 (0.92-1.01)	0.128	0.99 (0.96-1.02)	0.656
Chronic pulmonary disease						
No	1.00		1.00		1.00	
Yes	0.79 (0.67-0.93)	0.005	0.85 (0.74-0.97)	0.019	0.94 (0.85-1.04)	0.183
Diabetes						
No	1.00		1.00		1.00	
Yes	0.97 (0.90-1.04)	0.404	0.99 (0.93-1.05)	0.758	0.98 (0.94-1.02)	0.304
HF						
No	1.00		1.00		1.00	
Yes	0.67 (0.57-0.78)	<0.001	0.74 (0.66-0.84)	<0.001	0.89 (0.81-0.98)	0.014
Kidney disease						
No	1.00		1.00		1.00	
Yes	0.63 (0.51-0.79)	<0.001	0.89 (0.76-1.03)	0.106	0.71 (0.60-0.84)	<0.001
Private insurance						
No	0.84 (0.79-0.88)	<0.001	0.85 (0.81-0.89)	<0.001	0.98 (0.95-1.01)	0.124
Yes	1.00		1.00		1.00	

RR = Risk ratio; 95% CI = 95% confidence interval;

HF = heart failure; MI = myocardial infarction; SES = socio-economic status.

^(a)^most disadvantaged; ^(b)^least disadvantaged; ^(c)^MI type was based on ICD-10-AM coding terminology.

### Sensitivity analyses

Table 4 shows that alternative adjusted RRs for 28-day events using the two other definitions of Aboriginal status were similar to those for Model 4 in Table 2. Adjusted RRs for Aboriginal status based on 28- and 90-day events were also similar.

**Table 4:** Sensitivity analysis using (i) three definitions for Aboriginal identification, and (ii) 28-day vs 90-day events

	**IHD**		**MI**	
**Method of identifying Aboriginal status**	**RR (95% CI)**	**p value**	**RR (95% CI)**	**p value**
**(i) Coronary angiography**				
≥25% of admissions: 28-day event	0.94 (0.86-1.03)	0.186	0.94 (0.86-1.03)	0.195
First admission for event: 28-day event	0.97 (0.88-1.06)	0.473	0.96 (0.88-1.06)	0.431
Any admission: 90-day event	0.94 (0.86-1.02)	0.119	0.96 (0.89-1.04)	0.371
**(ii) Transfer to metropolitan hospital**				
≥25% of admissions: 28-day event	0.96 (0.88-1.04)	0.305	0.98 (0.91-1.05)	0.579
First admission for event: 28-day event	0.97 (0.90-1.06)	0.535	0.99 (0.92-1.07)	0.888
Any admission: 90-day event	1.00 (0.93-1.07)	0.942	1.00 (0.94-1.07)	0.958
**(iii) Receipt of coronary angiography if transferred to metropolitan hospital**				
≥25% of admissions: 28-day event	0.98 (0.93-1.04)	0.569	0.97 (0.91-1.03)	0.275
First admission for event: 28-day event	1.02 (0.94-1.05)	0.900	0.97 (0.91-1.04)	0.423
Any admission: 90-day event	0.95 (0.90-1.03)	0.061	0.97 (0.92-1.03)	0.338

Results are compared to Model 4 from Table 2 which has been adjusted for age groups, sex, residential area, SES, IHD category (for IHD) or MI type (for MI), 5-year history of chronic pulmonary disease, diabetes, HF, kidney disease and private insurance.

RR = Risk ratio; 95% CI = 95% confidence interval; IHD = ischaemic heart disease; MI = myocardial infarction; SES = socio-economic status; HF = heart failure.

## Discussion

Our study expands on other Australian studies investigating Aboriginal disparities in receipt of coronary angiography by restricting the analysis to rural patients and separating the effects of transfer to metropolitan hospitals from receipt of coronary angiography *per se*. After adjusting for age and sex, Aboriginal people presenting to rural hospitals with acute IHD were less likely to be transferred to metropolitan hospitals and if transferred were also less likely to receive coronary angiography. These disparities were mainly explained by the higher prevalence of comorbidities and to a lesser extent by the lower rate of private insurance among Aboriginal people.

Rural WA experienced 5,540 acute IHD events (average 3 events/day) in 2005–09 with Aboriginal people over-represented and their profile being consistent with previous Australian studies [9,10,12]. Our analyses investigated patients with a principal discharge diagnosis of IHD (which encompasses acute coronary syndrome (ACS) patients) as well as those with a principal discharge diagnosis of MI to increase our specificity. Our results were similar for IHD and MI events and our discussion is focused on MI events.

The proportion of patients who received coronary angiography in our study was higher than that recently reported by Randall *et al.*[10] for both Aboriginal (65.5% v 48.5%) and non-Aboriginal people (69.5% v 54.3%). These differences likely stem from our case selection since we used MI as the principal diagnosis only whereas Randall used MI in principal diagnosis or in second and third diagnoses along with IHD as the principal diagnosis. We found no Aboriginal disparity in receiving coronary angiography when unadjusted but observed significant disparities after adjusting for age and sex which is a reflection of the substantial different age and sex profiles of Aboriginal and non-Aboriginal MI patients [3]. The results of our full model are consistent with Randall’s study [10] where an Aboriginal disparity was found in receiving coronary angiography after adjusting for age, sex, admission year, AMI type and admitting hospital (adjusted hazard ratio 0.81, 95% CI 0.74-0.88), which was explained by the higher burden of comorbidities and lower rate of private insurance among Aboriginal people. Similarly, Ranasinghe *et al.*[12] and Roe *et al.*[28] did not find any Aboriginal disparity in their multivariate models, although the latter study was probably under-powered due to small numbers of Aboriginal people. On the other hand, Coory *et al.*[9] found disparities for coronary revascularisation (for which coronary angiography is a pre-requisite) amongst Aboriginal people after adjusting for age, sex, SES, remoteness, hospital characteristics and comorbidities (RR 0.78, 95% CI 0.64-0.94). Whereas Coory’s study was a person-based analysis looking at first-ever admission for MI in the 5-year period, ours was an events-based analysis; the former being useful for focussing on outcomes while the latter is more useful for health services/management decisions and planning. Restricting our analyses to first-ever MI in the 5-year period produced similar results (Model 6 in Additional file 2). The differences between our and Coory’s study may reflect state differences (WA v Queensland) or the time period examined (2005–2009 v 1998–2002) with possible improvement in Aboriginal care more recently.

Our study adds to those of Randall [10], Ranasinghe [12] and Coory [9] by separating from coronary angiography the effects of transfer to a metropolitan hospital (where coronary artery procedures are performed) as the disparity in receiving coronary angiography among rural WA patients could reflect inequity in their transfer to metropolitan hospitals [29]. Therefore, we restricted the analysis to rural patients whereas earlier studies controlled for remoteness of hospitalisation. Aboriginal people of the same demographic profile and MI type were less likely to be transferred to a metropolitan hospital, and when transferred were less likely to receive coronary angiography. These disparities were accounted for by comorbidities (like HF and kidney disease which are associated with adverse outcomes) and private insurance. Although we did not adjust for all comorbidities and risk factors (alcohol, smoking), inclusion of the Charlson comorbidity score and these additional risk factor variables did not change the RR for Aboriginal people (Model 7 in Additional file 2). Our results support those of the Australian and New Zealand SNAPSHOT ACS study [30] in that the burden of comorbidities accentuates the challenges faced in applying evidence-based guidelines among patients in this context.

Patients without private insurance were 16% less likely to receive coronary angiography than those with private insurance. Notably, among demographically and clinically similar patients without private insurance, there was no significant difference between Aboriginal and non-Aboriginal patients in the likelihood of receiving coronary angiography. However, Aboriginal people were substantially less likely to have private insurance than non-Aboriginal people (5.8% versus 32.2%). The effect of private insurance may be two-fold. Firstly, it provides the option of metropolitan transfer to the private hospital system if public beds are not available. Patients in the private system are more likely to receive coronary artery procedures than those in the public system [31,32]. This accords with the anecdotal reports of rural doctors regarding the difficulties they encounter in transferring non ST-elevation MI patients to public metropolitan hospitals, with some of these patients being transferred to the private system if they have private insurance. Secondly, given that we used an ecological measure for SES, private insurance may in fact be a proxy for individual-level SES or educational attainment. Thus, privately insured patients are more affluent, educated, articulate and possibly more likely to demand a transfer to a metropolitan hospital for appropriate care than less affluent patients [33,34]. Private insurance was not a factor in receiving coronary angiography once the patient had been transferred, suggesting that the private insurance-associated disparity in receiving coronary angiography is mediated through patient transfer to metropolitan hospitals.

One of the strengths of this study was the use of person-linked hospital and mortality data with state-wide coverage which allowed us to follow patients throughout the 28-day event. Without such access to person-linked hospital data, we would not be able to determine if the patient had received coronary angiography as this procedure is recorded as a separate admission. Our use of 28-day events was based on previous studies and ICD-10 coding standards [17-19], but repeating the analysis for 90-day events produced similar results, showing that the RRs are relatively robust with respect to the definition of an event.

The limitations of our study are those inherent with reliance on routinely collected administrative data. For example, the absence of individual-level SES data (especially in rural areas where large, heterogeneous areas can be allocated the same SES), more specific clinical information on severity of MI (although HMDC records MI as transmural and subendocardial, providing some indication of severity) and patient decisions to refuse further treatment limits our understanding of these disparities. Aboriginal people may refuse transfer to a metropolitan hospital based on negative experiences of family and friends. In fact, many Aboriginal people, particularly those from remote areas, find tertiary hospitals unwelcoming and are reluctant to attend for diagnosis [15,16]. As we did not have access to either the patient’s residential address or hospital name (confidentiality considerations), we could not adjust for the distance of patients’ residence from the hospital. Restricted to WA data only, it was possible to overlook patients who were transferred to other states for further treatment, in particular in far North WA where transfer to Royal Darwin Hospital is possible. Although Aboriginal status is under-reported in administrative data, our sensitivity analyses with three methods of Aboriginal identification produced similar results suggesting that our findings are robust with respect to Aboriginal identification.

## Conclusion

Our findings question the way in which Australia’s universal health insurance scheme is operating to support access of rural residents, especially Aboriginal people, to life-saving treatments. This disparity is particularly relevant in those with MI because the current National Heart Foundation of Australia/Cardiac Society of Australia and New Zealand guidelines [35] recommend coronary angiography in all patients with MI unless there are specific contraindications. Rural residents would benefit from consistent state-wide guidelines, protocols and processes for the management of ACS and system-wide coordination and integration of patient transfer (including central referral coordinating unit and clearly defined roles and responsibilities of individual health professionals) [36]. Our results suggest that urban health practitioners and policy makers can continue to claim that they treat Aboriginal and non-Aboriginal people alike based upon clinical indications, as private insurance is acting as an effective filter to reduce rural residents (where a greater proportion of Aboriginal people live) accessing interventional cardiology. If health practitioners and policy makers are truly committed to reducing health disparities, they must reflect upon the broader systems in which disparity is perpetuated and work towards a systems improvement [37].

## Abbreviations

ACS: Acute coronary syndrome; ARIA+: Accessibility Remoteness Index of Australia Plus; CARP: Coronary artery revascularisation procedures; HF: Heart failure; HMDC: Hospital Morbidity Data Collection; ICD-10-AM: International Classification of Diseases Australian Modification 10^th^ revision; IHD: Ischaemic heart disease; MI: Myocardial infarction; RR: Risk ratios; SEIFA: Socio-Economic Indexes for Areas; SES: Socio-economic status; WA: Western Australia.

## Competing interests

The authors declare that they have no competing interests.

## Authors’ contributions

DL, JMK, FMS, SCT, TGB, MSTH and PLT were involved in the concept and design of this study. DL extracted the linked data and performed the statistical analyses, with statistical advice from MWK. DL, JMK, FMS, MWK, JAW and TGB interpreted the results. DL constructed the figure and tables, and, initiated and coordinated the write-up. All authors read and approved the final manuscript.

## Pre-publication history

The pre-publication history for this paper can be accessed here:

http://www.biomedcentral.com/1471-2261/14/58/prepub

## Supplementary Material

Additional file 1 Full model RR in MI patients for coronary angiography, transfer, and coronary angiography if transferred. Click here for file

Additional file 2 Ratio of Aboriginal to non-Aboriginal risks of coronary angiography, transfer, and coronary angiography if transferred. Click here for file
