# Supplementary material for: Transfers to metropolitan hospitals and coronary angiography for rural Aboriginal and non‐Aboriginal patients with acute ischaemic heart disease in Western Australia
Source: BMC Cardiovasc Disord. 2014 May 1;14:58. doi: 10.1186/1471-2261-14-58 (PMC4021447; doi:10.1186/1471-2261-14-58)
Supplement: Additional file 1 — Full model RR in MI patients for coronary angiography, transfer, and coronary angiography if transferred. [file 1471-2261-14-58-S1.pdf]

Additional file 1 Full model RR in MI patients for coronary angiography, transfer, and coronary angiography if transferred

	Coronary angiography		Transfer to metropolitan hospital		Coronary angiography if transferred to metropolitan hospital	
	RR (95% CI)	p value	RR (95% CI)	p value	RR (95% CI)	p value
Aboriginal status						
Non-Aboriginal	1.00		1.00		1.00	
Aboriginal	0.97 (0.87-1.07)	0.510	1.00 (0.92-1.08)	0.939	0.98 (0.91-1.05)	0.546
Age groups						
25-34 years	1.12 (0.95-1.32)	0.186	1.06 (0.93-1.21)	0.376	1.06 (0.96-1.16)	0.250
35-44 years	1.02 (0.93-1.12)	0.734	1.04 (0.96-1.13)	0.302	0.98 (0.93-1.04)	0.478
45-54 years	1.00		1.00		1.00	
55-64 years	0.94 (0.86-1.03)	0.163	0.99 (0.93-1.07)	0.864	0.95 (0.90-0.99)	0.048
65-74 years	0.80 (0.71-0.90)	<0.001	0.82 (0.74-0.91)	<0.001	0.97 (0.91-1.03)	0.314
75-84 years	0.48 (0.40-0.58)	<0.001	0.55 (0.47-0.64)	<0.001	0.87 (0.80-0.96)	0.005
Sex						
Male	1.00		1.00		1.00	
Female	0.95 (0.87-1.04)	0.313	0.96 (0.89-1.03)	0.260	0.98 (0.94-1.03)	0.523
Residential area						
Regional	1.00		1.00		1.00	
Remote	1.01 (0.92-1.10)	0.875	1.05 (0.98-1.13)	0.146	0.96 (0.91-1.01)	0.121
SES quartiles						
1st quartile[a]	1.00		1.00		1.00	
2nd quartile	1.12 (1.02-1.24)	0.020	1.10 (1.00-1.20)	0.030	1.02 (0.97-1.07)	0.438
3rd quartile	1.12 (1.01-1.24)	0.036	1.10 (1.01-1.21)	0.036	1.01 (0.95-1.07)	0.686
4th quartile[b]	1.11 (0.99-1.25)	0.076	1.16 (1.06-1.27)	0.002	0.95 (0.88-1.03)	0.220
MI type[c]						
Transmural	1.00		1.00		1.00	
Subendocardial/other	0.97 (0.90-1.04)	0.348	0.96 (0.90-1.02)	0.177	1.01 (0.97-1.05)	0.712
Chronic pulmonary disease						
No	1.00		1.00		1.00	
Yes	0.78 (0.64-0.94)	0.009	0.84 (0.72-0.99)	0.032	0.92 (0.82-1.04)	0.185
Diabetes						

No	1.00			1.00			1.00	
Yes	0.94 (0.86-1.03)	0.195		0.96 (0.89-1.04)	0.372		0.97 (0.92-1.03)	0.306
HF								
No	1.00			1.00			1.00	
Yes	0.64 (0.53-0.78)	<0.001		0.72 (0.62-0.84)	<0.001		0.88 (0.78-0.99)	0.029
Kidney disease								
No	1.00			1.00			1.00	
Yes	0.62 (0.48-0.80)	<0.001		0.90 (0.76-1.06)	0.213		0.69 (0.56-0.84)	<0.001

RRs are presented for patients without private insurance

RR=Risk ratio; 95% CI=95% confidence interval;

HF=heart failure; MI=myocardial infarction; SES=socio-economic status

(a) most disadvantaged; (b) least disadvantaged; (c) MI type was based on ICD-10-AM coding terminology
