# Supplementary material for: Transfers to metropolitan hospitals and coronary angiography for rural Aboriginal and non‐Aboriginal patients with acute ischaemic heart disease in Western Australia
Source: BMC Cardiovasc Disord. 2014 May 1;14:58. doi: 10.1186/1471-2261-14-58 (PMC4021447; doi:10.1186/1471-2261-14-58)
Supplement: Additional file 2 — Ratio of Aboriginal to non-Aboriginal risks of coronary angiography, transfer, and coronary angiography if transferred. [file 1471-2261-14-58-S2.pdf]

Additional file 2 Ratio of Aboriginal to non-Aboriginal risks of coronary angiography, transfer, and coronary angiography if transferred

	**IHD**			**MI**	
Aboriginal status=Yes	RR (95% CI)	p value		RR (95% CI)	p value
**(i) Receipt of coronary angiography**					
Model 6	1.01 (0.92-1.11)	0.779		0.95 (0.87-1.04)	0.254
Model 7	1.00 (0.92-1.09)	0.959		0.98 (0.90-1.06)	0.621
**(ii) Transfer to metropolitan hospital**					
Model 6	1.03 (0.95-1.12)	0.431		0.99 (0.92-1.07)	0.790
Model 7	1.01 (0.93-1.09)	0.854		1.00 (0.93-1.07)	0.903
**(iii) Receipt of coronary angiography if transferred to metropolitan hospital**					
Model 6	0.98 (0.94-1.03)	0.514		0.95 (0.90-1.01)	0.125
Model 7	0.99 (0.94-1.04)	0.787		0.98 (0.93-1.04)	0.583

Model 6: adjusted for age group, sex, residential area, SES, IHD category (for IHD) or MI type (for MI), 5-year histories of chronic pulmonary disease, diabetes, HF, kidney disease and private insurance but restricted to first event in 2005-09.

Model 7: adjusted for age group, sex, residential area, SES, IHD category (for IHD) or MI type (for MI), 5-year histories of chronic pulmonary disease, diabetes, HF, kidney disease, private insurance, Charlson comorbidity score, smoking and alcohol.

RR=risk ratio with reference group being non-Aboriginal patients; 95% CI=95% confidence interval; HF=heart failure; IHD=ischaemic heart disease; MI=myocardial infarction; SES=socio-economic status
